# Stellate Ganglion Blockade and Left Cardiac Sympathetic Denervation With Left Stellate Ganglionectomy in a Patient With Refractory Electrical Storm: A Case Report

**DOI:** 10.7759/cureus.9098

**Published:** 2020-07-09

**Authors:** Nformbuh V Asangmbeng, Dominiq Okoduwa, Erskine A James

**Affiliations:** 1 Department of Internal Medicine, Navicent Health/Mercer University School of Medicine, Macon, USA; 2 Department of Cardiology, Navicent Health/Mercer University School of Medicine, Macon, USA

**Keywords:** ganglionectomy, ganglion blockade, electrical storm, ventricular tachycardia (vt) storm, ventricular fibrillation (vf) storm

## Abstract

Electrical storm (ES) is classified as at least three episodes of ventricular tachycardia (VT) or ventricular fibrillation (VF) in any 24-hour period. Stellate ganglion blockade and left stellate ganglionectomy have shown benefit in terminating ES. A 64-year-old white male with a past medical history of atrial fibrillation, coronary artery disease requiring previous cardiac bypass surgery in 1997, and coronary artery stents in 2003 presented with syncope and refractory ventricular tachycardia/fibrillation. He eventually underwent both an ultrasound-guided left stellate ganglion block and left cardiac sympathetic denervation with left stellate ganglionectomy. In the setting of refractory ES, the left stellate ganglion block followed by left stellate ganglionectomy can be a lifesaving intervention.

## Introduction

In a cardiac sense, an electrical storm (ES) is classified as at least three episodes of ventricular tachycardia (VT) or ventricular fibrillation (VF) in any 24-hour period [1]. The most common cause of ES is fibrosis (scar tissue) secondary to prior myocardial infarction (MI). Other causes include acute ischemia, decompensated heart failure, and electrolyte disturbances. ES can occur in as many as 10% to 20% of patients with automatic implantable cardioverter defibrillators (AICDs) placed for secondary prevention of dangerous cardiac arrhythmias and is associated with significant morbidity and mortality [2]. Patients with ventricular arrhythmias due to underlying heart structural disease are at the highest risk of developing ES, with ES being an independent predictor of mortality. This is especially true during the first few months following AICD implantation [1]. Due to the large sympathetic surge associated with ES, resistance to antiarrhythmic drug therapy can arise, making sympathetic blockade (including stellate ganglionectomy) necessary in some patients [3]. We present a patient with ES who underwent a left stellate ganglion block (LSGB) under ultrasound guidance, followed by left cardiac sympathetic denervation (LCSD) with a left stellate ganglionectomy. Catheter ablation for ES was not attempted in the patient, and therefore, the article will focus mostly on the medical utility of LSGB and LCSD with left stellate ganglionectomy.

## Case presentation

A 64-year-old white male with a past medical history of atrial fibrillation, coronary artery disease requiring previous cardiac bypass surgery in 1997, and coronary artery stents in 2003 was brought in by ambulance. Upon evaluation by emergency medical services (EMS) personnel, he was found to have atrial fibrillation with a rapid ventricular response. The patient’s heart rhythm later deteriorated to VF and torsades de pointes. He was cardioverted back to sinus rhythm, started on an amiodarone drip, and brought to the emergency department. On the day of admission, the patient had a total of nine cardiac arrests with VT and VF storms. These occurred while he was heavily sedated and on the ventilator. He required more than 30 defibrillations. In addition, he required vasopressor support with norepinephrine and vasopressin due to cardiogenic shock. After a successful return of spontaneous circulation (ROSC), the patient underwent emergent cardiac catheterization. He was found to have graft disease in both the circumflex and right coronary systems, with the distal vessels being non-revascularizable. The left internal mammary artery graft to the left anterior descending artery was patent. Echocardiography showed an ejection fraction of 60% - 65% with mild valvular heart disease. Due to cardiogenic shock, an intra-aortic balloon pump was placed via the right common femoral artery.

**Figure 1:**
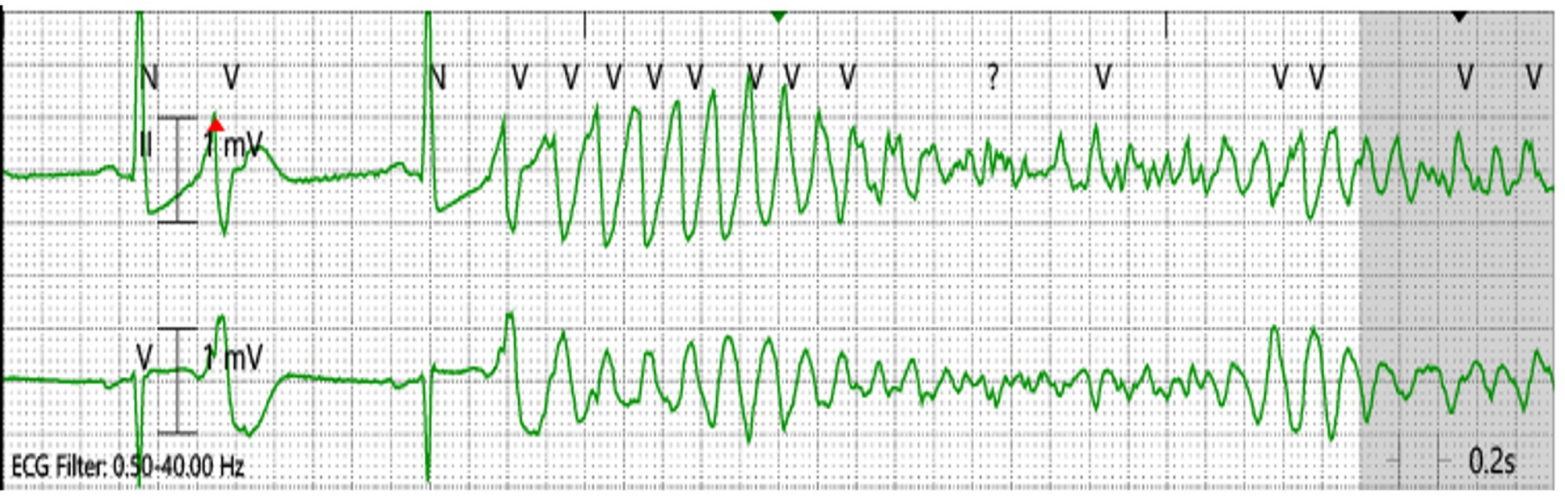
Telemetry showing ventricular fibrillation (VF) on the first day of admission

**Figure 2:**
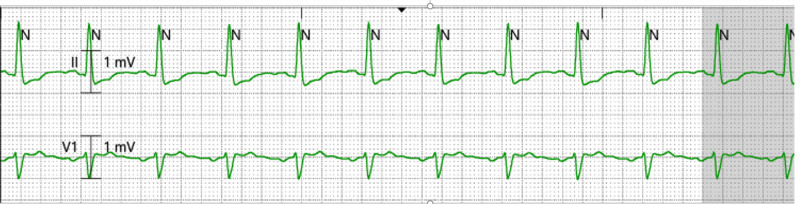
Telemetry showing return to normal sinus rhythm after ultrasound-guided left stellate ganglion block

After unsuccessful attempts to terminate the VT/VF with antiarrhythmic medications and cardiac defibrillation, the patient eventually had an ultrasound-guided left stellate ganglion block, which terminated the refractory ES, with no complications. Figures 1-2 show cardiac tracings before and after the procedure. Since the stellate ganglion block is usually temporary, lasting for hours to weeks, Cardiothoracic Surgery (CTS) was also consulted to perform a left cardiac sympathetic denervation with left stellate ganglionectomy.

Left stellate ganglion block procedure

After obtaining informed consent from the patient’s next of kin, the patient was placed in the supine position. The left neck was sterilized with sterile prep and draped. The anesthesiologist identified Chassaignac's tubercle, the skin was anesthetized with 1% lidocaine, and then an ultrasound-guided stimulating needle (22 Ga x 5 cm) was inserted to the C7 transverse process, above the Collis Longus muscle. After negative aspiration, a mixture of 15 ml of 0.5% ropivacaine with 4 mg of dexamethasone was slowly injected. The procedure was completed with no apparent complications, and the patient had a positive Horner's sign. The procedure resulted in the resolution of ES, as illustrated in Figure 2.

Left cardiac sympathetic denervation with left stellate ganglionectomy procedure

The patient was placed in the supine position. General anesthesia was successfully obtained by double-lumen endotracheal intubation. The patient was then placed in the right lateral decubitus position. The left chest was prepped and draped in the usual sterile fashion. Ventilation to the left lung was stopped by anesthesia. Cardiothoracic surgery attempted a video-assisted thoracoscopic sympathectomy. However, due to adhesions and an obstructed view, the procedure was stopped, and the decision was made to proceed with thoracotomy.

A left posterior muscle-sparing thoracotomy incision was made. The incision was extended down to the level of the auscultatory triangle. The fourth interspace was identified, and the chest was entered at that level. Upon entering the chest, the pleura overlying the 4th rib was incised using a cautery device. The sympathetic chain was identified at this level. The chain was then unroofed, both using sharp and blunt dissection, up to the level of the 2nd rib. The sympathetic chain was then excised using both cautery and sharp dissection up to the level of the 2nd rib. The pleura was opened more proximally using sharp dissection, and the sympathetic chain was dissected up toward the ganglion, again using sharp dissection. The sympathetic ganglion on the left was then identified. The branches of the sympathetic chain into the chest were identified. The stellate ganglion was divided in half just above these thoracic branches, using sharp dissection. The specimen was then passed off the field and sent to pathology. A fresh-frozen analysis revealed ganglion cells at the resected portion of the stellate ganglion. The patient tolerated the procedure well. There were no complications.

Throughout his hospital course, the patient received amiodarone, lidocaine (which was weaned and switched to mexiletine), and isoproterenol. A cardiac electrophysiologist was consulted, and ultimately, an AICD was placed. Eventually, after several weeks in the hospital, the patient was hemodynamically stable and free of further episodes of VT/VF. He was discharged home.

## Discussion

Stellate ganglion blockade and left stellate ganglionectomy have been shown to be very beneficial in terminating ES. The stellate ganglion is a collection or group of sympathetic nerve fibers that are located near the sixth and seventh vertebrae. These sympathetic fibers are anterior to the vertebrae and are responsible for innervating the arms, face, and chest. Stellate ganglion blockade involves the use of ultrasound to pharmacologically block the stellate ganglion. Using ultrasound during LSGB has been shown to greatly improve success and significantly decrease complications [4]. Often used in anesthesia, stellate ganglion blockade has been utilized for pain-relieving procedures applied to alleviate complex regional pain syndromes or pain secondary to diseases, such as shingles or vascular insufficiency. During an ES, it is thought that the propagation of catecholamine release from the aforementioned sympathetic fibers leads to further arrhythmias and subsequent catecholamine release [3]. Several studies have suggested and shown that a blockade of the stellate ganglion fibers has been useful in terminating the propagation of catecholamine release and improving potentially life-threatening arrhythmias which are refractory to antiarrhythmic drugs and defibrillations, as seen in ES [5].

Adjunct therapy to pharmacological stellate ganglion blockade has been the left stellate ganglionectomy. This procedure aims to remove the stellate ganglion and subsequently remove a major sympathetic innervation site of the heart. Access to the ganglion is often obtained through video-assisted thoracoscopic surgery (VATS). Ganglion denervation can also be obtained through a supraclavicular incision. Once accessed, the sympathetic ganglion chain is then removed [3]. The denervation of sympathetic fibers leads to a decrease in catecholamine release and decreases the threshold for potentially fatal arrhythmias, such as VT. The denervation in a stellate ganglionectomy also has been shown to prolong the ventricular refractory phase [6]. The efficacy of ganglionectomy has been demonstrated in subpopulations where catecholamines were suspected to potentiate adrenergic-mediated arrhythmias. For example, Wilde et al. demonstrated the efficacy of stellate ganglionectomies in teens who suffered from catecholaminergic polymorphic VT. In these individuals, it is thought that catecholamine release from the stellate ganglion can precipitate deadly arrhythmias [6]. In individuals with the condition, beta-blockers can be used, but some individuals remain refractory to this therapy. Wilde et al. demonstrated that ganglionectomies provide therapeutic intervention and decrease the likelihood of potentially life-threatening arrhythmias. Although more invasive, the use of the left stellate ganglionectomy, like the ganglion blockade, has been proven to help prevent catecholamine surges, thereby preventing ventricular arrhythmias that are refractory to defibrillation and antiarrhythmic medications and that, consequently, can be fatal.

## Conclusions

Although not commonly performed, LSGB and LCSD can be lifesaving treatment modalities. They are effective methods of treating ES-associated catecholaminergic surges resistant to routine advanced cardiac life support procedures, such as defibrillations and anti-arrhythmic therapies. Due to the invasiveness of LCSD, performing LSGB prior to LSCD is very important in order to identify patients with ES who are likely to respond to LSCD. In addition, LSGB complications are reduced with ultrasound-guided techniques.
